# Circular RNA CDR1as promotes tumor progression by regulating miR-432-5p/E2F3 axis in pancreatic cancer

**DOI:** 10.1186/s12935-021-01812-3

**Published:** 2021-02-16

**Authors:** Xingcheng Xiong, Jiarui Feng, Xiao Yang, Hanjun Li, Qiao Shi, Jing Tao, Jian Chang

**Affiliations:** 1 Department of Pancreatic Surgery, Renmin Hospital of Wuhan University, 238 Jiefang Road, Wuhan, 430060 Hubei China; 2 Department of Medical Management, Renmin Hospital of Wuhan University, 238 Jiefang Road, Wuhan, 430060 Hubei China; 3 Department of Hepatobiliary Surgery, Qianjiang Central Hospital of Hubei Province, 22 Zhanghua Middle Road, Qianjiang, 433199 Hubei China; 4 Department of Hepatobiliary Surgery, Qianjiang Hospital Affiliated to Renmin Hospital of Wuhan University, 22 Zhanghua Middle Road, Qianjiang, 433199 Hubei China; 5 Department of Hepatobiliary Surgery, Qianjiang Clinical Medical College, Health Science Center, Yangtze University, 22 Zhanghua Middle Road, Qianjiang, 433199 Hubei China

**Keywords:** Pancreatic cancer, Circular RNA/miRNA/mRNA, Tumor progression, Early diagnose

## Abstract

**Background:**

Pancreatic cancer (PC), characterized with high growth rate and metastatic rate. It’s urgently necessary to explore new mechanism of PC. Circular RNA/miRNA/mRNA network was widely reported to participate in the cancer progression.

**Methods:**

In this research, circular RNA CDR1as (circCDR1as) was identified by microarray analysis and detected in pancreatic cancer (PC) tissues and cells. Transwell, colony-forming assay, nude mouse tumorigenicity assay were used to determine the function of circCDR1as in PC. Western blot, dual luciferase reporting test were applied to investigate the mechanism.

**Results:**

We found that circCDR1as was highly expressed in PC tissues. The levels of circCDR1as in PC tissues and cells were higher than those in controls. CircCDR1as promoted the migration, invasion and proliferation of PC cells in vitro and tumor growth in vivo via mediating E2F3 expression by sponging miR-432-5p.

**Conclusions:**

In conclusion, circCDR1as could promote the development of PC and might be a novel diagnostic target for PC.

## Introduction

Pancreatic cancer (PC) is becoming a leading cause of cancer death due to its rapid growth and high metastatic rate [1]. The incidence and mortality rate of PC has been increasing in recent years [2]. Most PC patients are diagnosed at late stage due to the lack of effective biomarkers [3]. The only effective treatment for PC patients is surgical resection, but 50−80% of patients will have recurrence of the disease one year after surgery [4]. Until now, the specific pathogenesis of PC is not clear. Therefore, searching for new target is urgently needed to promote early diagnosis and treatment of PC.

Circular RNAs are non-coding RNAs that form closed-loop RNA molecules by reverse shearing [5]. Circular RNAs function as miRNA sponges, RNA-binding protein sponges, gene transcription regulator and protein translator [6, 7]. Increasing evidence has shown that circular RNAs are aberrantly expressed in tumors and associated with tumor proliferation, migration, invasion, prognosis and chemoresistance, making them potential targets for cancer research [6, 8, 9]. In PC, how circular RNAs mediate the disease requires in-depth study.

E2F transcription factor 3 (E2F3) family, a transcription factor family, functions in cell cycle, cellular differentiation, proliferation and apoptosis [10, 11]. Research shows that E2F3 can contribute to tumor development via causing excessive cell proliferation and apoptosis [12]. In PC, Yang reported that E2F3 was involved in proliferation and invasion of PC cells [13]. Sun found that the up-regulation of E2F3 was associated with the PC development [14].

In this study, we selected circular RNA CDR1as (circCDR1as) as the research target and performed microarray analysis. We found that circCDR1as was highly expressed in PC tissues and cells. In addition, circCDR1as promoted the proliferation, migration and invasion of PC cells, which was closely related to miR-432-5p-mediated expression of E2F3. In vivo, circCDR1as knockdown reduced the tumor growth of PC. This work may provide new directions for the early diagnosis of PC patients.

## Materials and methods

### Pancreatic cancer patients and tissue samples

 Tumor tissues and matched normal tissues from 27 PC patients were collected immediately after pancreatectomy and stored at − 80 °C in Renmin Hospital of Wuhan University (Wuhan, Hubei, China). Their clinicopathologic features were shown in Table 1. This study was approved by the Renmin Hospital of Wuhan University ethics committee. All patients did not receive neoadjuvant therapy and signed the informed consent form.

**Table 1:** Clinicopathologic features of PC patients

Clinicopathologic features	No.
Gender	
Male	17
Female	10
Age (years)	
< 60	21
≥ 60	6
Tumor size	
≤ 5	11
> 5	16
TNM stage	
I–II	13
III–IV	14
Differentiation grade	
Well/moderately	20
Poorly/undifferentiated	7

### Cell culture

The human PC cell lines PC-3, PANC1, ASPC1, CFPAC-1, MIApaCa-2, BXPC-3 and normal pancreatic ductal epithelial cell line HPDE6-C7 were purchased from American Type Culture Collection (ATCC, Manassas, VA, USA) and cultured in DMEM or RPMI1640 medium (Sigma-Aldrich, USA) containing 10% fetal bovine serum, 1% penicillin and streptomycin (Sigma-Aldrich, USA). HPDE6-C7 cell is normally cultured in keratinocyte serum-free (KSF) medium supplemented by epidermal growth factor and bovine pituitary extract. All cells were cultured at 37 °C in 5% CO_2_.

### Cell transfection

Sh-circCDR1as, OE-circCDR1as, miR-432-5p mimic and inhibitors were obtained from GenePharma (Shanghai, China). All vectors were transfected into PANC1 and ASPC1 cell lines using lipofectamine 2000 (Invitrogen, Carlsbad, USA) in accordance with the manufacturer’s instructions.

### Microarray analysis

Total RNA was extracted from 5 frozen PC tissues and matched normal tissues using TRIzol reagent (Invitrogen, Carlsbad, CA, USA), and then purified by the RNeasy Mini Kit (Qiagen, Valencia, CA, USA). Microarray assay was performed by the Agilent Array platform (Agilent Technologies) according to the user guidelines. The differently expressed genes in PC tissues were selected using the standard of p < 0.05 and fold-change > 2.

### Real‐time quantitative PCR

Total RNA was extracted from PC tissues or cells by TRIzol reagent (Life Technologies, Carlsbad, USA). To detect the expression levels of circular RNAs CDR1as, miR-432-5p, and E2F3, real-time quantitative PCR was conducted, and GAPDH was used as the control. All primers were as follows: circCDR1as (forward 5′-GCTGATCTTCTGACATTCAGG-3′, reverse 5′-GAGTTGTTGGAAGACCTTGAC-3′); miR-432-5p (forward 5′-AACGAGACGACAGAC-3′; Reverse, 5′-CTTGGAGTAGGTCATTGGGT-3′); E2F3; GAPDH (forward 5′-ATGTTGCAACCGGGAAGGAA-3′, reverse 5′-AGGAAAAGCATCACCCGGAG-3′).

### Colony formation assay

PANC1 and ASPC1 cells pretreated with different vectors were inoculated in 6-well plates, incubated in an incubator (37 °C, 5% CO_2_) for 10 days and fixed in methanol. Afterwards, 0.1% crystalline violet (Sigma-Aldrich, USA) staining was performed. The colonies were observed and stained under the microscope after 3 times of PBS rinsing.

### Cell growth assay

Cell growth was evaluated by Counting kit‑8 (CCk‑8, Dojindo Molecular Technologies) in accordance with the manufacturer’s instructions. Cells were seeded at 1 × 10³ cells/well in 96‑well plates. Then, CCk‑8 solution was added at 24 h, 48 h, 72 h and 96 h. The absorbance was measured at wavelength of 450 nm using a microplate reader (Thermo Fisher Scientific).

### Transwell assay

300 µl of PANC1 and ASPC1 cell cultures were seeded into the upper chamber of the transwell chamber (8 µm in size, Corning, NY, USA), and 700 µl of complete medium was added to the lower chamber. After 24 h of incubation at 37 °C in 5% CO_2_, the cells were fixed and stained with 4% paraformaldehyde and 0.1% crystalline violet (Sigma-Aldrich, USA). Then the number of invading cells was observed under the optical microscope.

### Flow cytometry

Cells were digested with 0.25% EDTA-free trypsin. Then the culture solution of cells each well was collected and having fallen off cells after digestion in a flow tube. Dyeing FITC and PI by Annexin V-labelled detection kit (Life technologies, USA) according to manufacturer’s protocol. After 15 mins of incubation in the dark, the rate of apoptosis was detected by flow cytometer (BD Biosciences).

### Dual luciferase reporting test

By investigating the Encyclopedia of RNA Interactomes (http://starbase.sysu.edu.cn/) database, we obtained binding sites between miR-432-5p and circCDR1as or E2F3. The circular RNA CDR1as and E2F3 sequences containing the miR-432-5p binding sites were inserted into the luciferase reporter vectors (WT-circCDR1as, WT-E2F3). The site-specific mutation of the miR-432-5p binding sites in the circCDR1as and E2F3 sequences were also inserted into the luciferase reporter vectors (MUT-circCDR1as, MUT-E2F3). PANC1 and ASPC1 cells were co-transfected with miR-432-5p mimic or NC mimic with WT-circCDR1as/MUT-circCDR1as, WT-E2F3/MUT-E2F3 using Lipofectamine 2000. Finally, the luciferase activity was detected 24 h after transfection using Dual-Luciferase Reporter Assay System (Promega, Madison, WI, USA).

### Western blot

Total proteins were extracted from pancreatic cancer cells using RIPA buffer (Sigma-Aldrich, USA). After SDS-PAGE electrophoresis, the proteins were transferred to the PVDF membranes. Then, the membranes were incubated with primary antibody (anti-GAPDH, anti-E2F3) overnight at 4 °C, followed by incubation of second antibodies at room temperature for 1 h. All samples were subjected to chemiluminescence (Bio-Rad, USA) imaging system according to instructions.

### Vivo experiments

 The animal experimental procedures of this study followed the guidelines of the Renmin Hospital of Wuhan University Animal Ethics Committee and were approved by the Animal Care and Use Committee. PANC1 and ASPC1 cells (0.1 ml, 2 × 10^7^/ml) were injected into the peritoneal cavity of BALB/c nude mice (n = 12, Changzhou Cavins Laboratory Animals Co., Ltd.) after cells were stimulated with sh-cirCDR1as or sh-NC dividely, and each group was consisted of 3 mice. After 2 weeks, when the tumor was visible to the naked eye, the size of the tumor was measured by caliper once a week. The tumor volume was calculated by the following formula: volume = 0.5 × length × width^2^.

### Statistical analysis

All biological experiments were repeated at least three times and the results were expressed as mean ± standard deviation and analyzed using Graphprism software. T-test was used for differences between groups. p < 0.05 represented a significant difference.

## Results

### CircCDR1as was highly expressed in PC tissues and cells

**Fig. 1:**
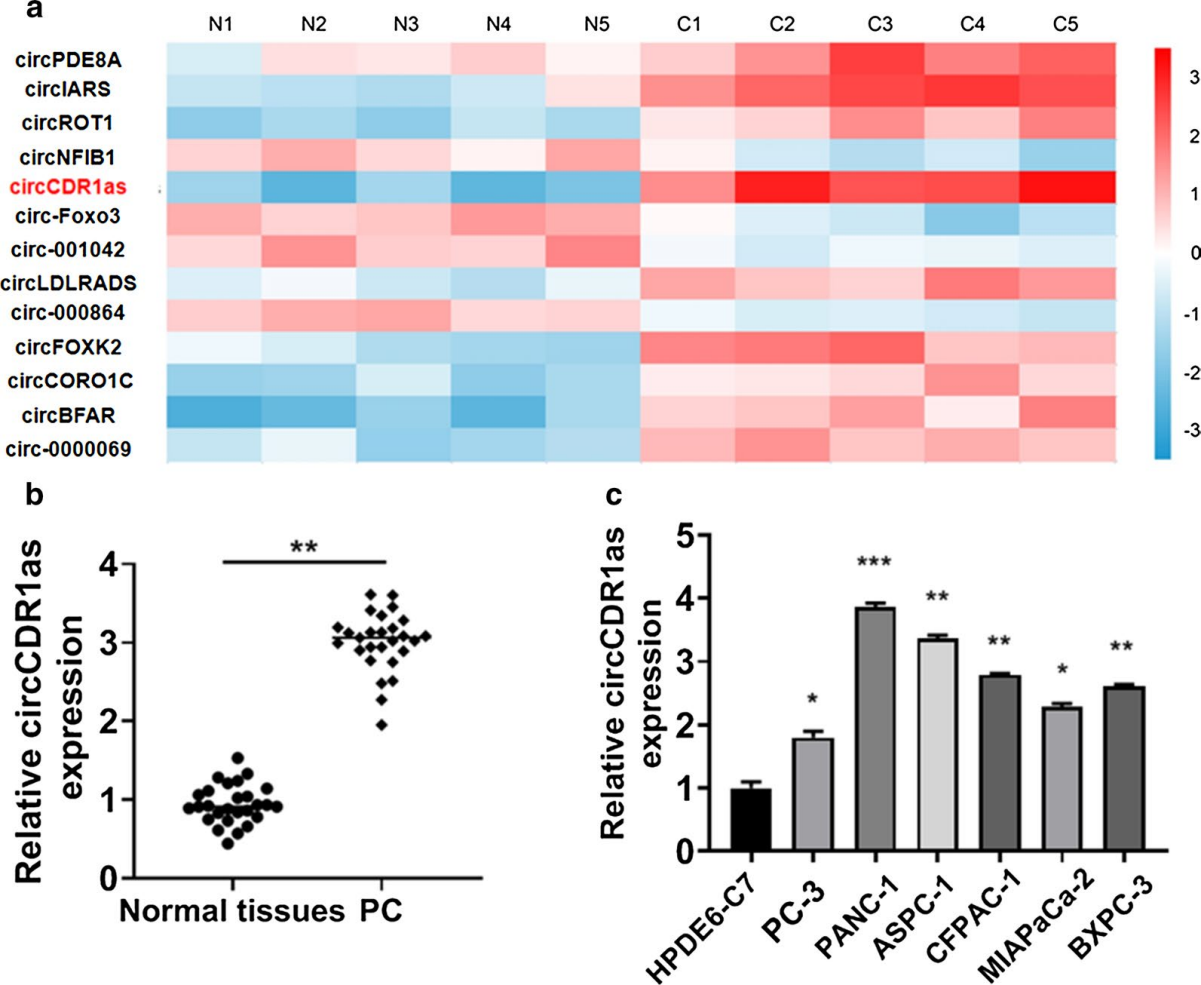
CircCDR1as expression is up-regulated in pancreatic cancer (PC) tissues and cells. **a** CircCDR1as was highly expressed compared with other circular RNAs according to microarray analysis. **b** Relative expression of circCDR1as in normal tissues and pancreatic cancer tissues. **c** The expression of circCDR1as in human pancreatic cancer cell lines (*p < 0.05, **p < 0.01, ***p < 0.001 )

Microarray analysis of PC tissues revealed that the expression of circCDR1as was the highest compared with other 12 circular RNAs (Fig. 1a). The detection of circCDR1as expression in 27 pairs of PC tissues and matched normal tissues showed that circCDR1as expression was up-regulated in PC (Fig. 1b). In addition, circCDR1as expression was higher in PC cell lines than normal pancreatic ductal epithelial cell line HPDE6-C7 (Fig. 1c). These findings suggested that circCDR1as was abnormally expressed in PC.

### CircCDR1as knockdown inhibited the metastasis and proliferation, promoted apoptosis of PC cells

**Fig. 2:**
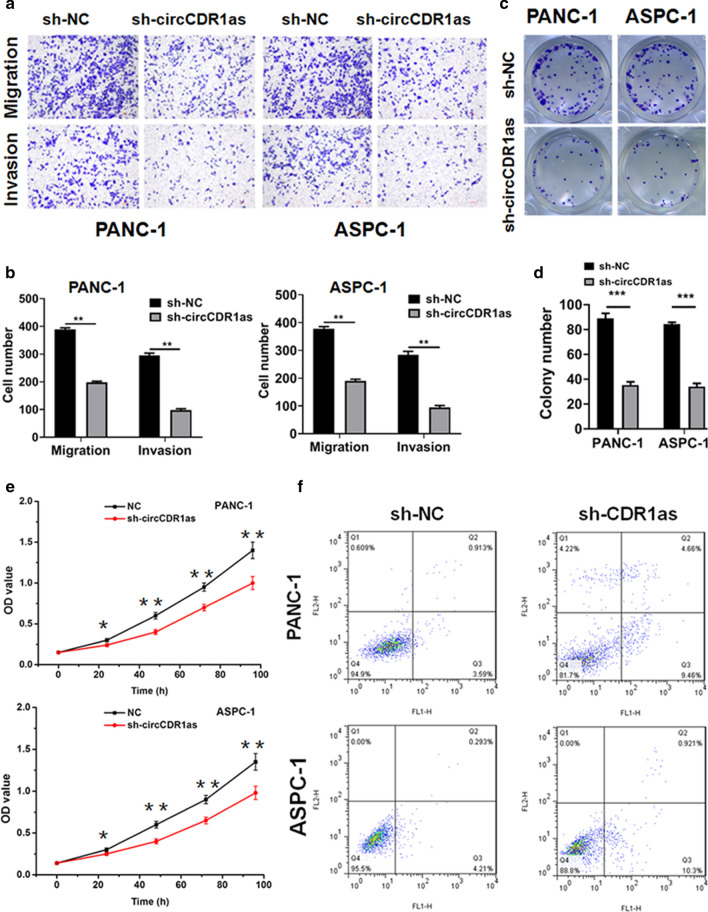
CircCDR1as induces pancreatic cancer cells migration, invasion and proliferation. **a**,** b** The migration and invasion of pancreatic cancer cells transfected with sh-NC and sh-circCDR1as by transwell assay. **c**, **d** The proliferation of pancreatic cancer cells transfected with sh-NC and sh-circCDR1as. **e** CCK-8 assay measured the cell viability of pancreatic cancer cells transfected with sh-NC and sh-circCDR1as. **f** The apoptosis of pancreatic cancer cells transfected with sh-NC and sh-circCDR1as (*p < 0.05, **p < 0.01, ***p < 0.001)

PANC-1 and ASPC-1, two cell lines with the highest circCDR1as expression, were selected to evaluate the effect of circCDR1as on PC. Inhibiting circCDR1as significantly suppressed the migration and invasion of PC cells (Fig. 2a, b). In addition, inhibiting circCDR1as significantly decreased the proliferation amounts of PC cells (Fig. 2c, d). The inhibition of circCDR1as significantly decreased the viability of PANC-1 and ASPC-1 cells, promoted apoptosis of PANC-1 and ASPC-1 cells (Fig. 2e, f). The above results indicated that circCDR1as could induce the metastasis and proliferation of PC cells, but inhibit apoptosis of PC cells.

### CircCDR1as acted as a sponge for miR-432-5p

**Fig. 3:**
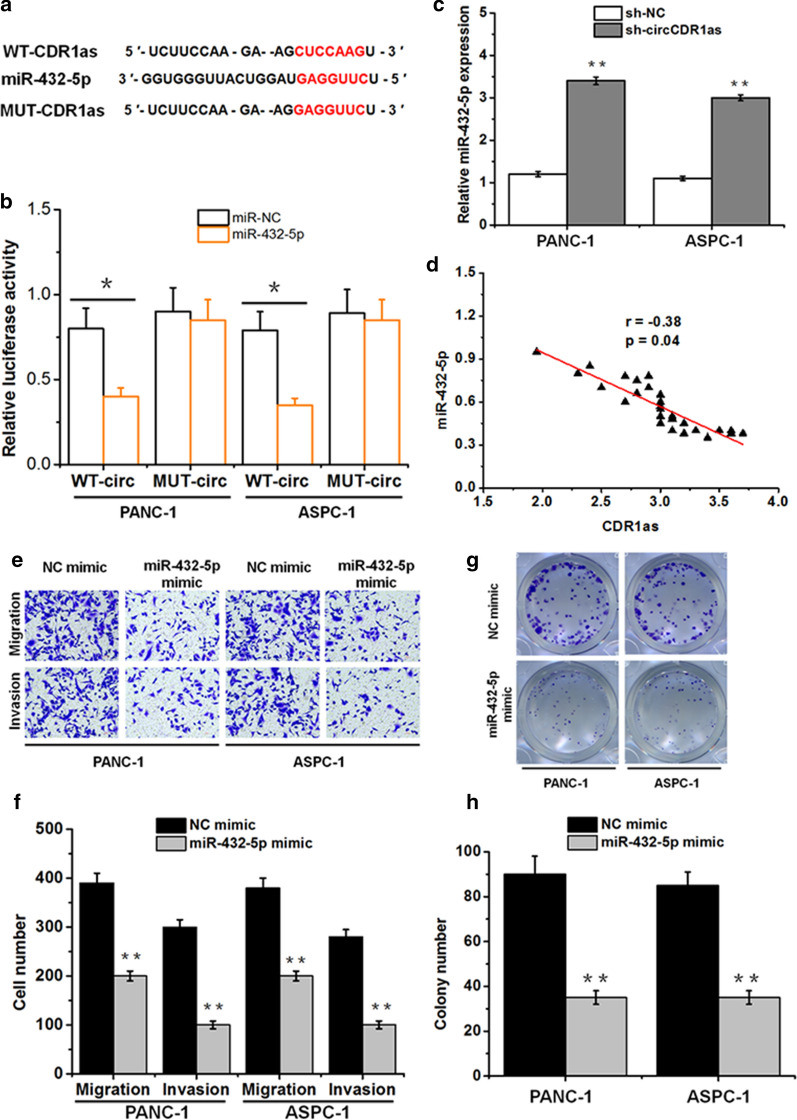
MiR-432-5p targets circCDR1as in pancreatic cancer cells. **a** The predicted binding site of circCDR1as and miR-432-5p by Starbase database. **b** The relative luciferase activities of wild-type and mutated circCDR1as 3ˊ-UTR luciferase vectors co-transfected with control or miR-432-5p mimics in PANC-1 and ASPC-1 cells. **c** Relative expression of miR-432-5p in normal tissues and PC tissues. **d** The relation of circCDR1as expressions and miR-432-5p expressions in PC tissues. **e**,** f** The migration and invasion of PANC-1, ASPC-1 cells transfected with NC mimic and miR-432-5p mimic. **g**,** h** The proliferation of PANC-1, ASPC-1 cells transfected with NC mimic and miR-432-5p mimic (*p < 0.05, **p < 0.01)

The signaling pathway of circCDR1as in PC cells was explored since circRNAs was reported to sponge miRNAs to take part in tumor development. By Starbase database, the possible binding sites between circCDR1as and miR-432-5p were found (Fig. 3a). To testify the interaction between circCDR1as and miR-432-5p, dual luciferase reporting test was performed, showing that luciferase activity was significantly reduced in PC cells stimulated with WT-circCDR1as and miR-432-5p, but there were no evident differences in PC cells transfected with MUT-circCDR1as and miR-432-5p (Fig. 3b). We also found circCDR1as inhibition induced the expression of miR-432-5p in PC cells (Fig. 3c). The expression of circCDR1as and miR-432-5p was negatively related in 27 PC tissues (Fig. 3d). Over-expressing miR-432-5p in PC cells suppressed the migration, invasion and proliferation of cells (Fig. 3e–h). The above results showed that circCDR1as could sponge miR-432-5p in PC cells.

### E2F3 served as a target for miR-432-5p in PC cells

**Fig. 4:**
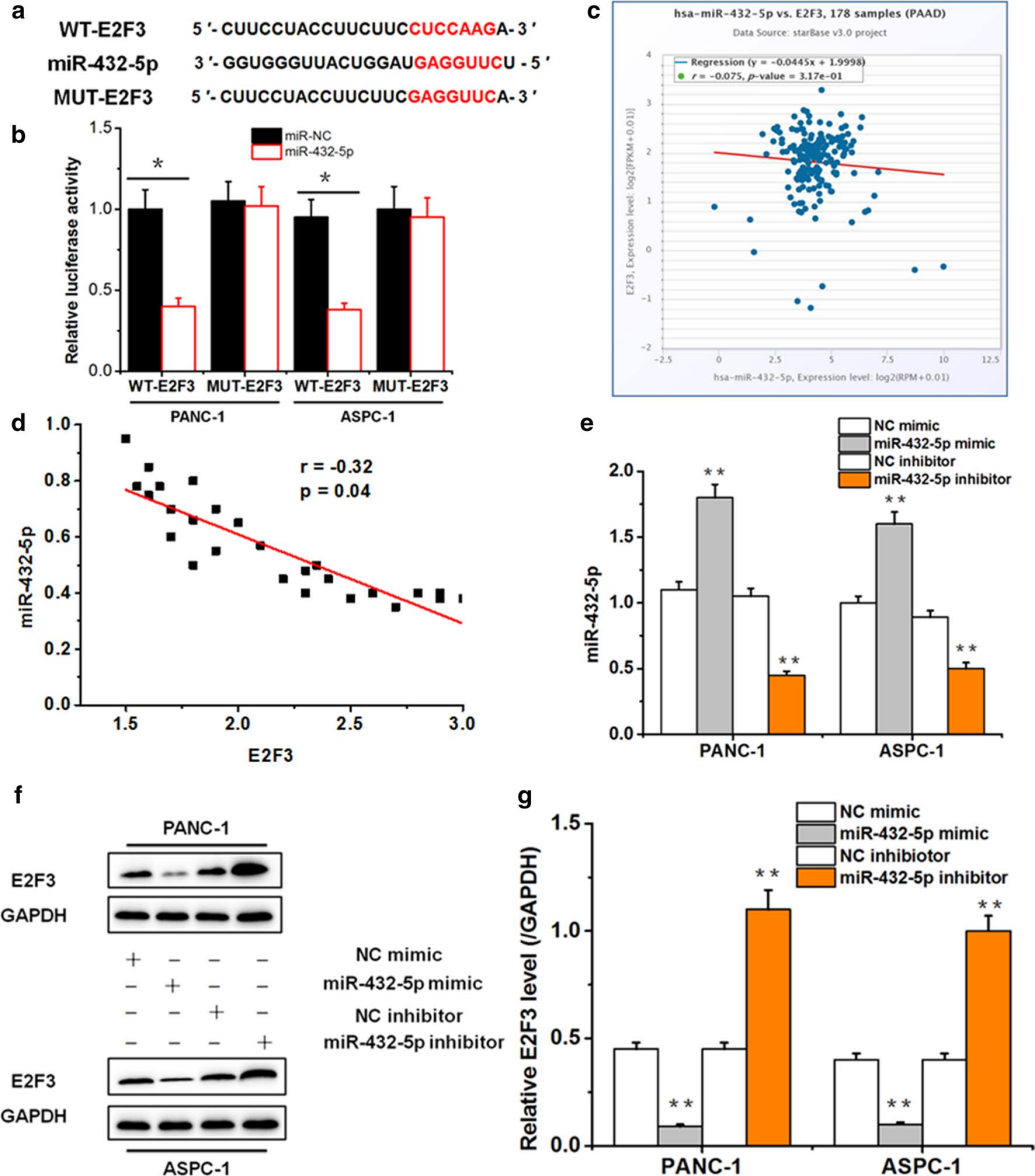
E2F3 targets miR-432-5p in PANC-1, ASPC-1 cells. **a** The predicted binding site of miR-432-5p and E2F3 by Starbase database. **b** The relative luciferase activities of wild-type and mutated E2F3 3′-UTR luciferase vectors co-transfected with control or miR-432-5p mimics in PANC-1 and ASPC-1 cells. **c** The relationship between the expression of miR-432-5p and E2F3 in pancreatic cancer samples by Starbase database. **d** The relation between the expression of miR-432-5p and E2F3 in PC samples in this study. **e** The miR-432-5p mRNA expression in PC cells transfected with NC mimic, miR-432-5p mimic, NC inhibitor, miR-432-5p inhibitor. **f**, **g** The E2F3 expression in PANC-1, ASPC-1 cells transfected with NC mimic, miR-432-5p mimic, NC inhibitor, miR-432-5p inhibitor via western blot (*p < 0.05, **p < 0.01)

By further study, we noticed that E2F3 possibly had reciprocity with miR-432-5p. The predicted binding sites between E2F3 and miR-432-5p were listed in Fig. 4a. Dual luciferase reporting test (Fig. 4b) proved that the luciferase activity of miR-432-5p significantly down-regulated in PC cells stimulated with WT-E2F3, but no significant differences of relative luciferase activity was found in PC cells transfected with MUT-E2F3. According to Starbase database, the concentration of E2F3 was negatively correlated with the concentration of miR-432-5p in PC (Fig. 4c), which was also showed in the detection of 27 PC tissues in this study (Fig. 4d). Furthermore, PC cells transfected with miR-432-5p mimic vector showed higher levels of miR-432-5p and lower levels of E2F3, but PC cells transfected with miR-432-5p inhibitor vector showed lower levels of miR-432-5p and higher levels of E2F3 by western blot (Fig. 4e–g). These findings suggested that miR-432-5p directly interacted with E2F3 in PC cells.

### CircCDR1as promoted PC progression via miR-432-5p/E2F3 axis in vitro and vivo

**Fig. 5:**
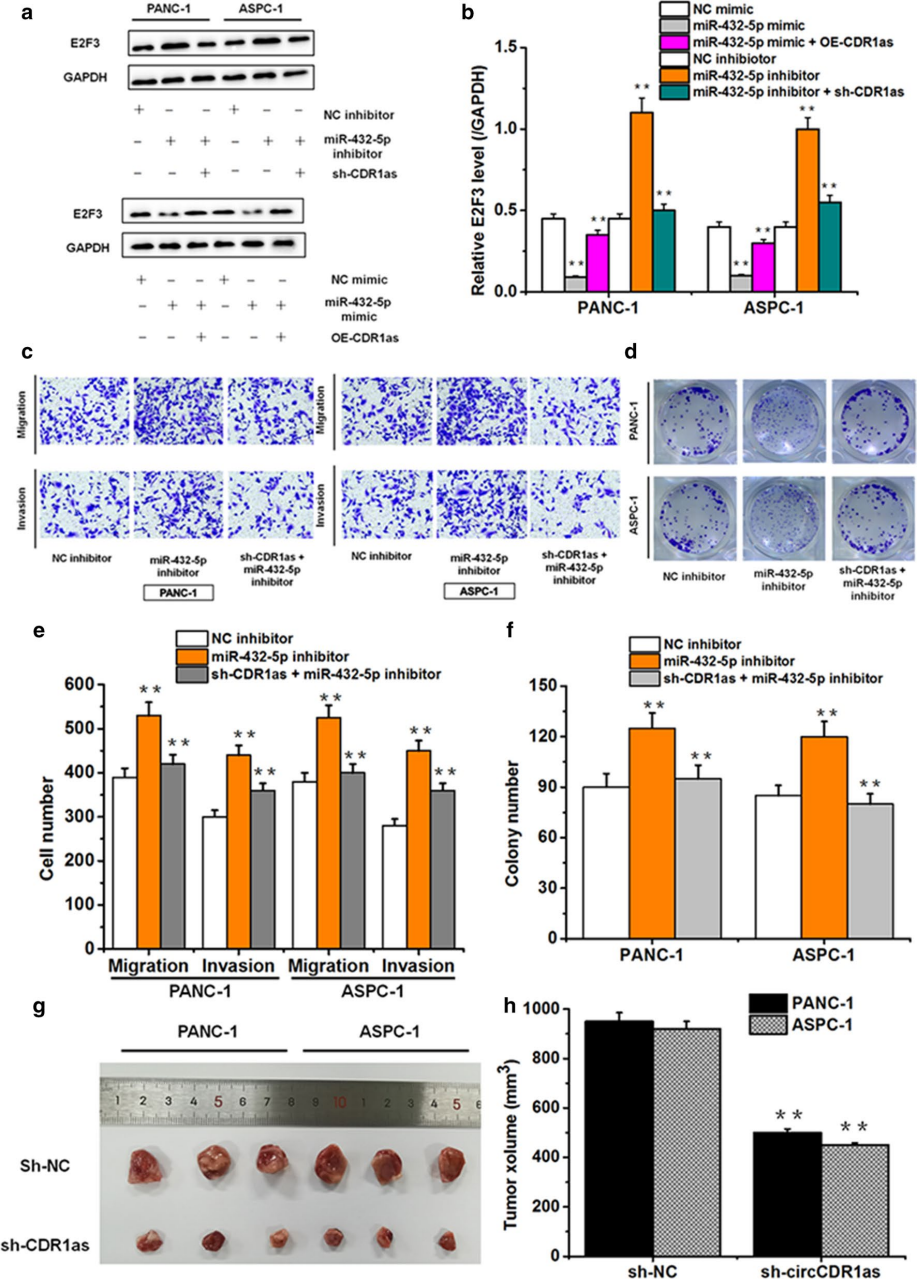
CircCDR1as promotes progression of pancreatic cancer by regulating E2F3 via miR-432-5p. **a**,** b** Western blot detected the expression of E2F3 in PANC-1, ASPC-1 cells transfected with NC inhibitor, miR-432-5p inhibitor, sh-circCDR1as + miR-432-5p inhibitor, NC mimic, miR-432-5p mimic and OE-circCDR1as + miR-432-5p mimic. **c**,** e** The migration and invasion of PANC-1, ASPC-1 cells transfected with NC inhibitor, miR-432-5p inhibitor, sh-circCDR1as + miR-432-5p inhibitor. **d**,** f** The proliferation of PANC-1, ASPC-1 cells transfected with NC inhibitor, miR-432-5p inhibitor and sh-circCDR1as + miR-432-5p inhibitor. **g**,** h** Knocking down of circCDR1as inhibited the growth of pancreatic cancer xenograft tumor (*p < 0.05, **p < 0.01)

It’ s found that miR-432-5p inhibition induced the expression of E2F3, which was reversed by extra sh-circCDR1as supplement, miR-432-5p over-expression inhibited the expression of E2F3, which was reversed by extra OE-circCDR1as supplement (Fig. 5a, b). And the inhibition of miR-432-5p also promoted the migration, invasion and proliferation of PC cells. However, PC cells co-transfected with sh-circCDR1as and miR-432-5p inhibitor mimics lightened the effect caused by suppressing miR-432-5p (Fig. 5c–f). In vivo experiments, we also found that circCDR1as inhibition significantly decreased the volumes of PC tumors (Fig. 5g, h). These results stated clearly that circCDR1as could promote the progression of PC.

## Discussion

The discovery of circular RNA provides a new research direction for cancer research. So far, circRNAs have been widely explored in gallbladder cancer, gastric cancer, liver cancer, lung cancer, and prostate cancer [15–18]. In our study, we found that circCDR1as was significantly up-regulated in PC tissues through microarray analysis. Exploration on PC tissues and cells showed that circCDR1as was abnormally expressed in PC. The knockdown of circCDR1as in PC cells inhibited the proliferation, migration and invasion of cells, indicating that circCDR1as might exert oncogenic effects on PC.

To investigate the mechanism of circCDR1as in PC, we conducted further research. CircRNAs as miRNA sponge could inhibit the expression of miRNA and regulate the expression of target genes, which in turn played an important role in diseases. Li et al. [19] found that the circular RNA MAT2B promoted glycolysis and dysregulation in hepatocellular carcinoma by acting on miR-338-3p under hypoxic stress. Bi et al. [20] found that the cyclic RNA ZKSCAN1 targeted miR-1178-3p to inhibit the development of bladder cancer and was a potential indicator of prognosis. Wang et al. [21] found that the cyclic RNA circCRIM1 inhibited lung adenocarcinoma invasion and metastasis, which was correlated with its regulation of miR-182 expression. The present study found that circCDR1as inhibited the expression of miR-432-5p in PC cells, and the effects on PC cells migration, invasion and proliferation induced by circCDR1as inhibition were consistent with the effects of miR-432-5p over-expression on PC cells. The above evidence suggested that circCDR1as could achieve oncogenic function by targeting miR-432-5p in PC.

It is known that miRNAs bind to mRNAs to regulate tumorigenesis. According to previous studies, miR-432-5p could target and regulate E2F3, playing an important role in breast cancer [22], nasopharyngeal cancer [23], osteosarcoma [24], melanoma [25], and gastric cancer [26]. We also found that 1miR-432-5p sponged and negatively regulated E2F3 in PC cells. However, whether the function of circCDR1as in PC was linked with miR-432-5p/E2F3 pathway was unclear. In the present study, we proved that circCDR1as inhibition could significantly down-regulate the elevated E2F3 expression caused by miR-432-5p suppression. And circCDR1as could regulate the migration, invasion and proliferation of PC cells via sponging miR-432-5p. In vivo, inhibition of circCDR1as could suppress the tumor growth in PC.

In summary, circCDR1as regulates E2F3 expression in PC through sponging miR-432-5p, thus promoting the progression of PC. Therefore, circCDR1as may be a potential target for PC diagnosis.

## Data Availability

All data analyzed in the study are included in this published article.
